# A Self-Assembling Ferritin Nanoplatform for Designing Classical Swine Fever Vaccine: Elicitation of Potent Neutralizing Antibody

**DOI:** 10.3390/vaccines9010045

**Published:** 2021-01-13

**Authors:** Zekai Zhao, Xinghua Chen, Yibao Chen, Hui Li, Kui Fang, Huanchun Chen, Xiangmin Li, Ping Qian

**Affiliations:** 1 State Key Laboratory of Agricultural Microbiology, Huazhong Agricultural University, Wuhan 430070, China; zekaizhao@yeah.net (Z.Z.); cxhdeyx129@163.com (X.C.); yibaochen@webmail.hzau.edu.cn (Y.C.); huilihzau@yeah.net (H.L.); fangkui1987@126.com (K.F.); liuchenxinxin@163.com (H.C.); lixiangmin@mail.hzau.edu.cn (X.L.); 2 College of Veterinary Medicine, Huazhong Agricultural University, Wuhan 430070, China

**Keywords:** ferritin nanoplatform, classical swine fever virus, E2, neutralizing antibody

## Abstract

Protein-based self-assembling nanoplatforms exhibit superior immunogenicity compared with soluble antigens. Here, we present a comprehensive vaccine strategy for displaying classical swine fever virus (CSFV) E2 glycoprotein on the surface of ferritin (fe) nanocages. An E2-specific blocking antibody assay showed that the blocking rates in *p*E2-fe/Gel02 (84.3%) and a half-dose cohort of E2-fe/Gel02 (81.9%) were significantly higher (*p* < 0.05) than that in a ferritin-free cohort of *p*E2/Gel02 (62.7%) at 21 days post immunization (dpi) in vivo. Furthermore, quantitation of neutralizing potency revealed that a highly significant difference (*p* < 0.001) was observed between the *p*E2-fe/Gel02 cohort (1:32, equivalent to live-attenuated strain C at 1:32) and the *p*E2/Gel02 cohort (1:4) at 21 dpi. Moreover, the innate immune cytokines of IL-4 and IFN-γ activated by the half-dose (20 μg) cohort of E2-fe/Gel02 were equivalent to those elicited by the full dose (40 μg) of purified E2 in the *p*E2/Gel02 cohort at most time points. In conclusion, we successfully obtained an antigen-displaying E2-ferritin nanoplatform and confirmed high ferritin-assisted humoral and cellular immunities. Our results provided a novel paradigm of self-assembling nanovaccine development for the defense and elimination of potentially pandemic infectious viral pathogens.

## 1. Introduction

Classical Swine Fever virus (CSFV), a member of the *Pestivirus* genus of the *Flaviviridae* family, is closely related to the viruses that cause bovine viral diarrhea in cattle and border disease in sheep (OIE, 2020). It causes the disease CSF, also known as hog cholera, which is a contagious viral disease of domestic swine and wild boar [1,2]. Furthermore, CSF is considered a transboundary animal disease by the Food and Agriculture Organization of the United Nations (FAO).

The structural glycoprotein E2 of CSFV produces a predominant and efficacious humoral immune response of neutralizing antibodies and is the main cellular immunity inducer of specific CD8+ effector cytotoxic T lymphocytes [3,4]. However, a CSFV lapinized vaccine C-strain against CSF globally lacks the capability of serological differentiation between infected and vaccinated animals (DIVA) [5]. Some studies have confirmed that the utilization of live-attenuated or inactivated disease-causing pathogen vaccines carries the risk of virulence instability and mortality after immunization [6,7]. Therefore, the secretion antigen E2 is an optimal candidate for the CSFV vaccine development.

Various strategies have focused on subunit antigens and virus-like particle vaccines, though with low productivity or severe side-effects [8]. There is increasing evidence that a nanoparticle (np) vaccine can play a major role in the resolution of disease epidemics [9,10]. Among them, the naturally self-assembling ferritin (fe) np composed of 24 identical polypeptides in well-ordered arrays has been highly utilized in eliciting a broadly neutralizing antibody [11,12]. To our knowledge, there has been no precedent that has applied a 24-unit self-assembling ferritin np platform into CSFV antigen display and vaccine development. Therefore, the displaying E2 on the surface of ferritin would be a future nanovaccine orientation for CSFV elimination.

Species barriers between domestic pigs and experimental rabbits have been overcome. The C-strain, which is adapted to rabbits (ATR) and live-attenuated in pigs, has the unique ability to replicate in rabbit spleen and lymph nodes, causing a fever response [13]. Several studies have demonstrated that the residues P108 and T109 on the E2 glycoprotein determine the C-strain’s ATR [13,14,15]. Thus, rabbits are susceptible animals with highly safe and cost-saving characteristics for real-time CSFV evaluation.

Our previous results indicated a ferritin nanoparticle vaccine for foot-and-mouth disease virus can provide partial protection in a mice model [16]. In this study, we addressed the formulation of long-lived humoral immunity to CSFV elicited by recombinant E2-ferritin hollow nanospheres. The baculovirus–insect cell expression vector system (BEVS) expressing the signal peptide (Sp) of the human CD5 leader sequence (hCD5L) has been proven to be an optimal selection mechanism for the production of “native” protein conformation [11,17]. *Helicobacter pylori* (*H. Pylori*) ferritin present a 24-subunit polymer, as described in other reports [18]. Thus, we fused the E2 to the ferritin np N-terminus, starting from Asp5, separated by a Gly-Ser-Gly (GSG) linker to facilitate structure folding. The designed antigen E2 of CSFV was exhibited on the surface of the ferritin nanoplatform and resulted in the improved immunogenicity and stability of this novel E2-ferritin nanoplatform vaccine. Importantly, the in vivo experiments in ATR illustrated that a sensitive and potent neutralizing antibody response was elicited by the E2-ferritin nanoplatform.

Taken together, our results confirm the impact of an effective humoral immune response elicited by the E2-ferritin nanoplatform, as quantified by a potent neutralization potency index, and describe the ascendancy of self-assembled 24-unit ferritin nanocarriers. The superior immunogenicity of the ferritin particulate antigen presentation highlights the need to focus future efforts on the development of broadly protective interventions to mitigate future CSFV pandemics.

## 2. Materials and Methods

### 2.1. Cell Lines and Virus Strains

Cell lines of pK-15 (porcine kidney) were cultured in high-glucose DMEM (Dulbecco’s modified Eagle medium, HyClone, Marlborough, MA, USA) supplemented with 10% (*v*/*v*) FBS (heat-inactivated fetal bovine serum, Gibco, Carlsbad, CA, USA) for CSFV amplification and neutralizing antibody titration; sF9 insect cell lines were maintained in FBS-free insect cell culture medium (Gibco, Carlsbad, CA, USA) in a 27 °C incubator (Thermo Fisher Scientific, Carlsbad, CA, USA) with 5% humidified CO_2_. The CSFV C-strain (GenBank accession no. AY805221.1) and Alfort187 strain (GenBank accession no. NC_038912.1) were maintained in our laboratory.

### 2.2. Plasmids Construction and Transfection

*H. Pylori* ferritin (GenBank accession no. NP_223316) was synthesized by Sangon Bio. (Shanghai, China) with a signal peptide of the human CD5 leader sequence [19]. The BEVS was purchased from Thermo Fisher Scientific (Product no. 10712024) and operated according to the manufacturer instructions. The extracted bacmids of E2-ferritin and ferritin were then transfected into SF9 cells using a Cellfectin II Reagent (Product no. 10362100, Gibco, Carlsbad, CA, USA). The 3-copy CSFV E2 recombinant baculovirus expression vector system (rBEVS) was described previously [20]. For detailed information on gene amplification, see Table 1.

### 2.3. Animal Study Design

Rabbits (*n* = 4, female, 1.5 kg/rabbit, specific pathogen free, SPF) were purchased and maintained at the Experimental Animal Center of Huazhong Agricultural University (HZAU). Animals were given the prime immunization at day 1 via intramuscular injection and then a booster at day 21 [21]. Animals were challenged with CSFV live-attenuated strain C (WINSUN BIO. Ltd., Guangdong, China) via intravenous injection at day 41 and sacrificed at the end point for measurements. The water or aqueous adjuvant Gel02 (Montanide, Seppic™, Castres, France) was emulsified with antigens to activate immunomodulatory function [22]. Moreover, to highlight the multimer of E2-ferritin np platform in the induction of NAb, the unpurified half-dose of E2-ferritin (20 μg/rabbit) was evaluated in an independent cohort.

### 2.4. Animal Warfare Statement

The operation was conducted according to the corresponding guidelines of experimental animal operation of HZAU; the board for laboratory animals of HZAU provided approval for this experience. The corresponding ethical approval code is No. 00184168.

### 2.5. CSFV Antibody Blocking ELISA

Serum samples were collected with 10-day intervals after immunization and 7-day intervals after challenge. CSFV-specific NAbs in sera were tested using a HerdChek Classical Swine Fever Virus Antibody Test kit (CSFV Ab) (Product no. 06-43230-04, IDEXX, Westbrook, ME, USA) according to the manufacturer’s instructions.

### 2.6. Blood and Spleen Sample Collection and RT-qPCR Analyses

Blood and spleen samples were collected from individual subjects for analysis of viremia and viral tissue load by absolute RT-qPCR at the end of the experiment. Serum samples were prepared by centrifugation and stored at −80 °C. For relative RT-qPCR analysis, the blood samples (0.1 mL/rabbit) were harvested at different time points. The TRI reagent (product no. T9424, Sigma-Aldrich, Shanghai, China) was used for RNA extraction after homogenization according to the manufacture’s instrument. The cDNA was synthesized by ReverTra Ace^®^ qPCR RT Mix (FSQ-101, TOYOBO life science, Osaka, Japan), and then processed to relative RT-qPCR using TIIV 7 RT-qPCR instrument (ABI bio, Carlsbad, CA, USA). Here, we set the PBS cohort as the control cohort.

For absolute RT-qPCR assay, a detailed assessment was performed as previously described [23]. Briefly, the gene of CSFV strain C 5′UTR was amplificated by PCR and then cloned into the pMD18-T vector (TAKARA, Kyoto, Japan) as standards. The standards’ copy numbers were calculated as (concentration)/(number of bases) × 9 ×10^11^. The efficiency of the qPCR amplification is 96% according to the standards’ amplification curve. The extracted RNA products were treated by DNase I (TAKARA, Kyoto, Japan) followed by cDNA synthesis using ReverTra Ace^®^ qPCR RT Mix. Here, we set the valid definition of viral load as 331.7 copies/μg total RNA for the Ct value less than 31 according to the manufacturer’s instructions of the THUNDERBIRD Probe qPCR Mix (QPS-101, TOYOBO, Osaka, Japan). The concentration of DNA or RNA was detected using spectrophotometer (nanodrop 2000, Thermo). All the plasmids were verified by Sanger sequencing (Sangon bio, Shanghai, China). The primers and specific probe for CSFV amplification were used in Table 1.

For relative RT-qPCR assay, the procedure was used as previously described [24]. Briefly, the DNA sequence was amplified using the SYBR Green Master Mix (A25742, ABI, Carlsbad, CA, USA). The primers of GAPDH (reference gene), IL-4, and IFN-gamma were adapted from rabbits’ nucleotides (Table 1). PCR amplification was performed as: 2 min at 50 °C, 2 min at 95 °C, and 40 cycles of 15 s at 95 °C, and 30 s at 60 °C. The relative gene expression was presented as the relative mRNA fold change using the 2^−ΔΔCt^ with GAPDH as the reference gene, and PBS cohort samples as control for normalization [25,26].

### 2.7. Confocal Microscopy

Sixty hours after infection, sF9 cells were imaged using an Alexa Fluor 488 (product no. A32723, Invitrogen, Carlsbad, CA, USA) to detect cellular E2-ferritin and ferritin np expression. Here, we applied anti-his tag mAb (product no. D291-3, MBL, Nagoya, Japan) and anti-CSFV E2 mAb (product no. 9011, MEDIAN, Gangwon-do, Korea) as primary antibodies. Images were acquired using a 63X objective on a Zeiss LSM510 instrument (Oberkochen, Germany).

### 2.8. Lymphocyte Proliferation Assay

PBMC were extracted by Peripheral Blood Lymphocyte Separator (kit; product no. P8760, Solarbio, Beijing, China) [27]. Cells were cultured in 96-well plates (Thermo Fisher, Carlsbad, CA, USA) with 10^4^ cells in 100 μL per well, and stimulated with 50 μg/mL concanavalin A (Sigma) or 20 μL of inactivated C-strain (10^5^ TCID_50_/_mL_). DMEM was used as the negative control. The plates were incubated at 37 °C for 20 h, and then 5 mg/mL methylthiazol tetrazolium (MTT, Solarbio, Beijing, China) was added for further incubation at 37 °C for 4 h. Then, 100 μL of 10% dimethyl sulfoxide (DMSO, Solarbio, Beijing, China) was added to stop the reaction. The stimulation index (SI) was calculated with the following formula: SI = (OD sample well−OD blank well)/(OD negative well−OD blank well) at OD_492_ with triple technical repeats.

### 2.9. Viral Neutralizing Assay

Serum (100 μL) was collected in each cohort and then 2-fold serial dilution for neutralizing antibody titration. The CSFV strain Alfort187 (200 TICD_50_, 100 μL, 1.1-genotype) was used for a potent and broadly neutralizing reaction with anti-CSFV E2 antibody. The serum–virus complex was mixed at 37 °C for 1 h and then added into pK-15 cells in the 96-well plate for 2 days. The neutralizing titer was calculated by the Reed–Muench assay with 4 repetitions using indirect immunofluorescence assay (IFA).

### 2.10. Histidine (His) Tagged Affinity Purification

The nanoparticles were bound and purified using an Ni-NTA affinity column [28]. The sample supernatant was loaded at a rate of 1.0 mL/min, and the column was washed with 5 column volumes (CV) of buffer A (0.1 M PBS, 300 mM NaCl, 5 mM imidazole) at 1 mL/min. In addition, a concentration gradient of imidazole (from 0 to 500 mM) was flowed through the column at 1 mL/min. Fractions corresponding to the major peak were collected and concentrated for SDS-PAGE and Western blotting analysis.

### 2.11. Statistical Analysis

Numeric data were shown as means ± SD; the significance of differences was assessed by one/two-way ANOVA. Data analyses and graphing were performed using IBM SPSS (Statistical Product and Service Solutions) Statistics (IBM, New York, NY, USA) and GraphPad Prism 8.0 (GraphPad Software, La Jolla, CA, USA). *p* < 0.05 was considered statistically significant.

## 3. Results

### 3.1. Internal Architecture and Intracellular Location of the Ferritin Nanoplatform

**Figure 1:**
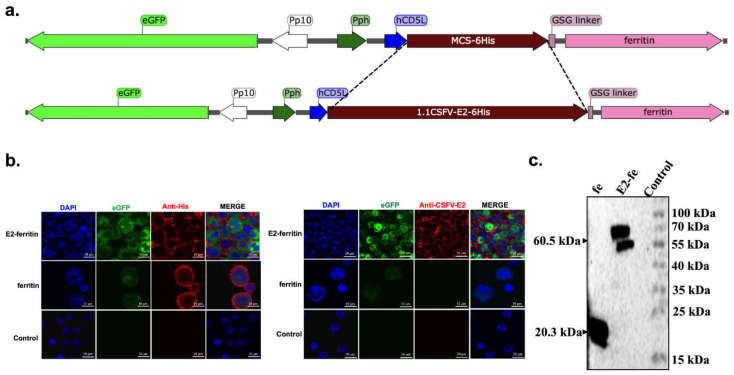
Characterization and intracellular localization of the E2-ferritin and ferritin nanoplatform. (**a**) Schematic representation of the ferritin (above) and E2-ferritin (below) nanoplatform expression cassette. (**b**) Immunofluorescence assay (IFA) for nanocage intracellular location using anti-His tag antibody (left) and anti-CSFV E2 mAb (right) as primary antibodies followed by Alexa Fluor 555 as secondary antibody using confocal microscopy. (**c**) Protein expression detected by Western blotting using anti-His tag monoclonal antibody.

To increase the exposure and delivery of the specific antigens, a universal ferritin nanoparticle platform was constructed, as shown in Figure 1a and Supplementary Materials, Figure S1. An enhanced green fluorescent protein reporter (eGFP) initiated by promotor P_10_ was inserted into the replication cassette as a transfection indicator. Furthermore, to explore the intracellular locations of E2 and ferritin, confocal microscopy was performed. The IFA results revealed an intense reaction with the antibodies against ferritin and CSFV E2, which were presented at the cytoplasm of sF9 cells (Figure 1b). With multiplicity of infection (MOI) of 1 at 3 days post infection (dpi), the immunoblotting results showed that the E2-ferritin and ferritin were successfully expressed after three rounds of stable passage (Figure 1c).

### 3.2. Amplification and Purification of the E2-Ferritin and Ferritin Np

**Figure 2:**
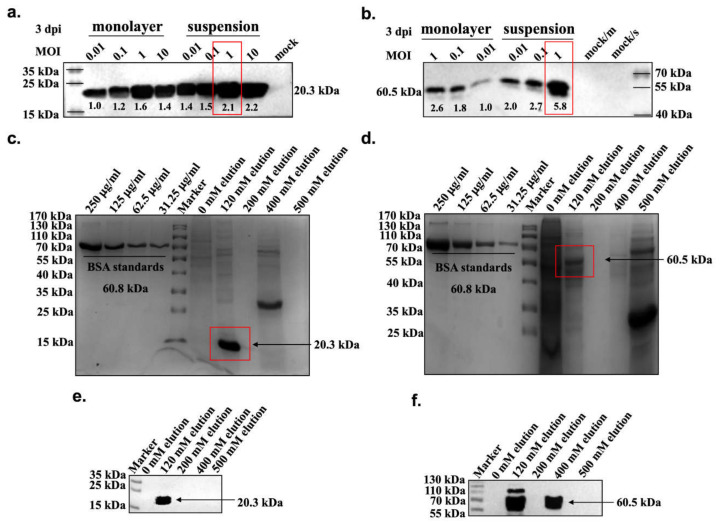
Amplification and purification of E2-ferritin and ferritin nanocarriers. The optimal proliferation conditions for ferritin in (**a**) and E2-ferritin in (**b**) np were both 1 MOI in suspension culture by Western blotting analysis. Protein density was measured by Image J software. The protein elution peak for ferritin in (**c**) and E2-ferritin in (**d**) were both in 120 mM imidazole by SDS-PAGE. Different concentrations of BSA were used as standard samples. The purified nanocages of ferritin in (**e**) and E2-ferritin in (**f**) were verified by immunoblotting using anti-His tag antibody. (For the uncropped blots of Figure 2a,b,e,f, see Supplementary Materials, Figures S2–S5).

To identify the efficient native function of the ferritin nanoplatform, E2-ferritin and ferritin np were processed in single-component extractions. Immunoblotting and protein band density analyses demonstrated that the E2-ferritin and ferritin exhibited sufficient expression at an MOI of 1 at 3 dpi (Figure 2a,b). The Western blotting results also confirmed that the nanoparticle components were cleaved efficiently between the Sp and the nanostructure under these conditions. As determined by SDS-PAGE analysis and Coomassie staining, the E2-ferritin and ferritin were highly pure (>95% purity) (Figure 2c,d). Both eluted solutions exhibited a single band of the 120 mM imidazole component on SDS-PAGE. In parallel, to confirm their identity, the eluted samples containing the ferritin-bound proteins were also analyzed by immunoblotting assay. The results showed that the two recombinant proteins had robust immunoreactivity with anti-His antibodies (Figure 2e,f).

### 3.3. The E2-Ferritin Nanospheres Efficiently Elicited Potent NAb In Vivo

The antiviral efficacy and neutralizing antibody inducing capacity of the E2-ferritin nanoplatform was assessed in an ATR model (Table 2). To further understand the role of ferritin in intercommunication with the immune system and the nature of the signals involved, a purified ferritin np cohort was designed in this experiment.

**Figure 3:**
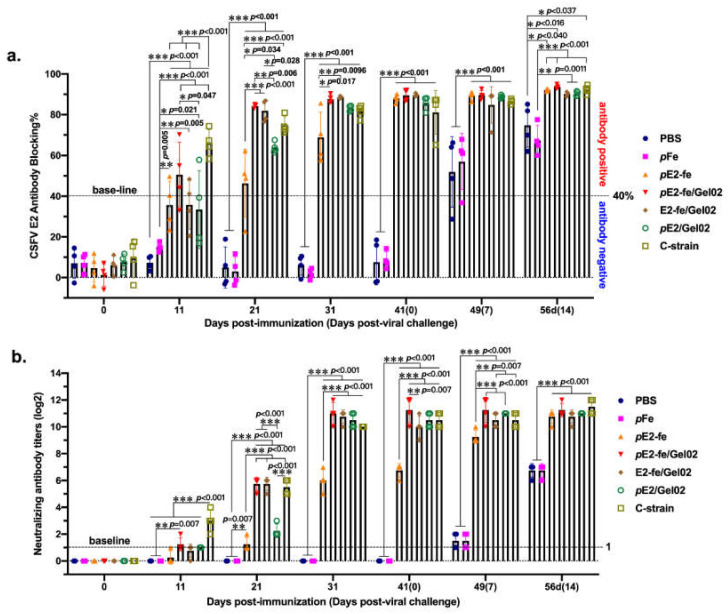
E2-specific antibody blocking analysis and neutralizing antibody titration in vivo. (**a**) CSFV E2-specific antibody blocking rate assay by IDEXX kit. (**b**) CAFV E2 neutralizing antibody titration in pK-15 cells. The data presented in this figure for (**a**,**b**) were calculated by two-way ANOVA analysis using GraphPad Prism 8.0 with 4 technical repeats. *, 0.05< *p* < 0.01; **, 0.01< *p* < 0.001; ***, *p* < 0.001.

First, serum CSFV antiviral antibody levels were measured by E2-recognition antibody-blocking ELISA (Figure 3a). At 11 dpi, compared with the non-E2 injection cohort of PBS (0/4) and fe (0/4), rabbits immunized with nanoparticle vaccines of *p*E2-fe (2/4), *p*E2-fe/Gel02 (3/4), E2-fe/Gel02 (2/4), subunit vaccine *p*E2/Gel02 (1/4), and the CSFV live-attenuated vaccine strain C (4/4) developed detectable antibody titers. The CSFV E2-specific antibody titer in the *p*E2-fe/Gel02 cohort was significantly higher than those in other groups, but was lower than that in the strain C cohort (*p* < 0.05). The results of the E2 antibody blocking ELISA showed that the blocking rates in the *p*E2-fe/Gel02 (84.3%) cohort and E2-fe/Gel02 (81.9%) cohort were higher than that in the *p*E2/Gel02 (62.7%) cohort at 21 dpi (*p* < 0.05). All E2-associated cohorts (88.0% of *p*E2-fe, 89.5% of *p*E2-fe/Gel02, 89.7% of E2-fe/Gel02, 85.7% of *p*E2/Gel02, and 81.1% of strain C) achieved peak levels at 41 dpi. No significantly E2-targeting antibodies in PBS and *p*fe groups were observed during the immunization.

The levels of CSFV-specific neutralizing antibodies produced by the four cohorts gradually increased (Figure 3b). Importantly, *p*E2-fe/Gel02, E2-fe/Gel02, and C-strain vaccinated rabbits were seroconverted at 21 dpi, with mean antibody-neutralizing rates of approximately 1:32, while *p*E2/Gel02 was 1:4 of the neutralizing titer. A highly significant difference (*p* < 0.001) was present between the *p*E2-fe/Gel02 and *p*E2/Gel02 cohorts at most time points.

### 3.4. The E2-Ferritin Nanocage Activated Sufficient Innate Immune Cytokine

**Figure 4:**
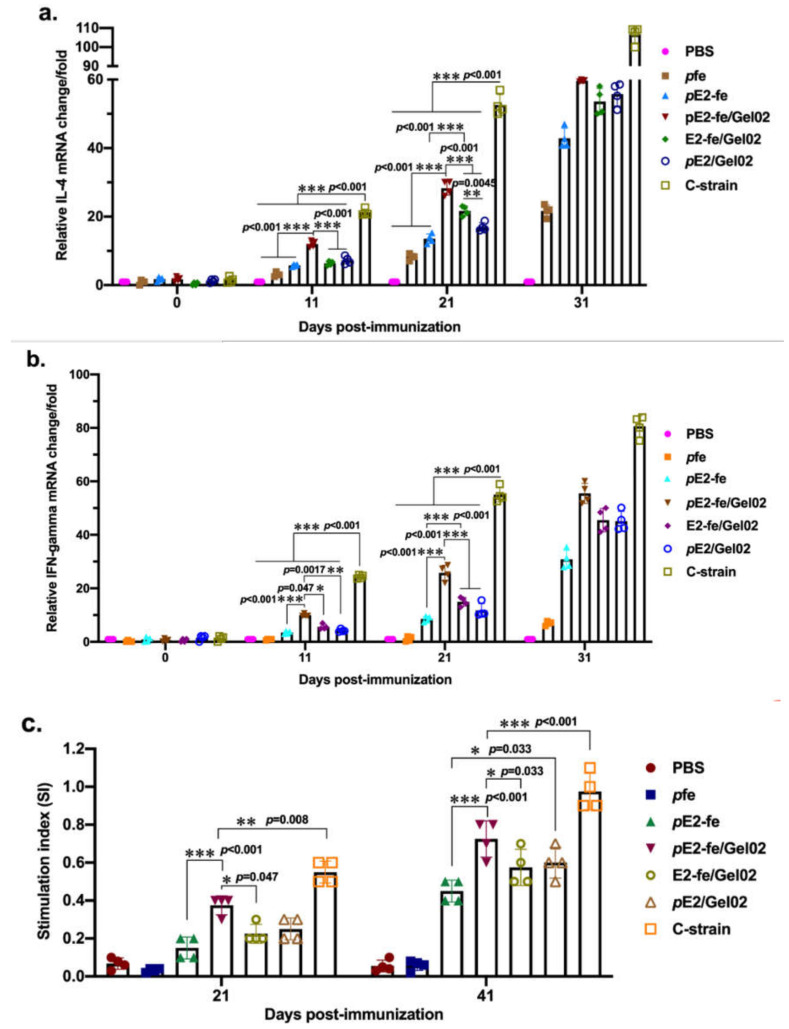
The humoral and cellular immunity response detection. (**a**) Relative IL-4 mRNA fold change detection by RT-qPCR. (**b**) Relative IFN-γ mRNA fold change detection by RT-qPCR. (**c**) Lymphocyte proliferation assay for cell viability determination via MTT. All the data in this figure are repeated 4 times. The data presented here were analyzed by two-way ANOVA assay using GraphPad Prism 8.0. *, 0.05 < *p* < 0.01; **, 0.01 < *p* < 0.001; ***, *p* < 0.001.

To comprehensively investigate the initiation of humoral and cellular immunity, the relative cytokine secretion levels of interleukin 4 (IL-4) and interferon-gamma (IFN-γ) were detected by real-time quantitative PCR (RT-qPCR) at days of 0, 11, 21, and 31 (Figure 4a,b). The results showed that the rabbits in the *p*E2-fe/Gel02 cohort triggered high levels of cytokines. Additionally, the relative IL-4 and IFN-γ mRNA fold change induced by the half-dose (20 μg) cohort of E2-fe/Gel02 were equivalent to those elicited by the full dose (40 μg) of purified E2 in the *p*E2/Gel02 cohort at most time points.

To explore immune system activation, we isolated peripheral blood mononuclear cells (PBMCs) from immunized experimental animals. The lymphocyte proliferation test demonstrated that the in vitro activation index for the E2-ferritin nanoplatform in the *p*E2-fe/Gel02 cohort was 2-fold higher than that in the *p*E2/Gel02 cohort at 21 dpi (Figure 4c).

### 3.5. The Novel E2-Ferritin Nanoplatform Vaccine Eliminated Viremia and Stereotyped Thermal Response in ATR

**Figure 5:**
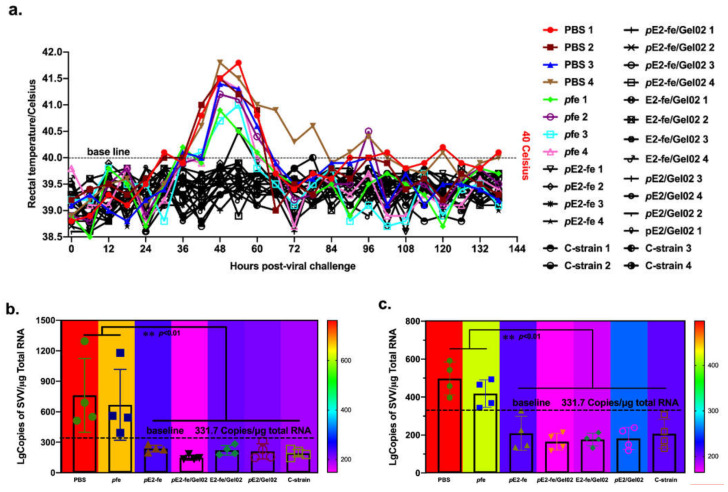
CSFV-specific stereotyped thermal response in ATR and viral tissue loading analysis. (**a**) Rectal temperature measurement and rabbits’ stereotyped thermal response identification post viral challenge. (**b**) CSFV viral tissue load analysis in spleen via absolute RT-qPCR. (**c**) Viremia analysis via absolute RT-qPCR. All the data showed in this Figure 5b,c are repeated 4 times. The data presented here were analyzed by two-way ANOVA assay using GraphPad Prism 8.0. **, 0.01 < *p* < 0.001.

To explore the efficacy of the ferritin nanoparticle vaccine in viral clearance, all experimental rabbits were inoculated intravenously with 1 dose of the CSFV C-strain. Data from the rectal temperature measurement indicated that the rabbit-specific stereotyped thermal responses were identified in the PBS cohort and fe np cohorts post viral challenge (Figure 5a). Specially, their temporary rectal temperatures exceeded 40 °C at 36–72 h post-viral challenge. Moreover, the E2-associated cohorts developed stably.

At 14 days post challenge (dpc), CSFV was detected in various organs of the rabbits by RT-qPCR, such as spleen and blood (Figure 5b,c). No viral RNA was detected in rabbits vaccinated with E2-interrelated cohorts and the CSFV C-strain cohort, whereas rabbits in the PBS and *p*fe cohorts had detectable viral RNA. Specifically, the valid definition of viremia was 331.7 copies/μg total RNA. In the viral spleen load and viremia studies, the highest viral RNA levels were detected in the PBS cohort (1294 and 592 copies/μg total RNA, respectively). The results demonstrated that the viremia and viral spleen loads in the PBS and *p*fe cohorts are significantly higher (*p* < 0.01) than that in the E2-associated vaccine cohorts.

## 4. Discussion

As the globally commercialized CSFV lapinized vaccine strain C failed in serological DIVA (differentiation between infected and vaccinated animals), the application of a novel ferritin nanoplatform with soluble E2 antigen provides a candidate for vaccine development for the eradication of the CSFV pandemic. Numerous self-assembling ferritin-associated studies showed that the exposed antigens or proteins were produced in native status and arranged in well-ordered manners, such as a bioluminescent tracer for liver cancer detection and receptor binding domain decoration for COVID-19 elimination [28,29]. Furthermore, the 24-subunit polymer was efficiently phagocytosed by body antigen recognition and presentation systems with a sensitive and potent neutralizing antibody response in vivo (see Figure 3a). All these key data concluded that the primary trial of the E2-ferritin nanocage strategy was highly successful.

Recently, several studies have demonstrated that domains A, B, C, and D of CSFV E2 glycoprotein cover all of the neutralizing peptides of therapeutic antigens [30]. A truncated E2 peptide consisting of neutralizing domains coated on an ELISA plate displayed a high sensitivity (119/127, 93.7%) and specificity (143/155, 92.3%), with agreements of 92.9% (262/282) and 92.2% (260/282) by the gold-standard of the IDEXX blocking ELISA kit, respectively [31]. Therefore, self-assembling or chimeric nanocages harboring different domains or structure-based truncated E2 subunits could maximize the expression of soluble protective immunogens. From the perspective of secreted proteins, the hCD5L Sp efficiently guided extracellular transportation, as demonstrated via Western blotting assay.

Typically, following an initial vaccination or infection, the rabbit’s adaptive immune system develops a suite of defenses, including memory B lymphocytes capable of developing sterilizing immunity or neutralizing antibodies targeted to bind to specific pathogens, and memory T lymphocytes that assist in regulating the immune reaction and inducing the cytotoxicity of infected cells [32]. In addition, the immune adjuvant Gel02 in this study exerted a strong immune-modulating effect. Cohorts between the single-component *p*E2-fe (without Gel02) and *p*E2-fe/Gel02 (with Gel02) were significantly different (*p* < 0.001). In this instance, an initial vaccination with a particular adjuvant (Gel02)-emulsified agent (E2-ferritin) engendered a sufficient adaptive immune response to confer sterilizing immunity.

Potent induction of adaptive immunity depends on efficient activation of the innate immune system. Cytokines such as IFN-γ and IL-4 play key roles in the development of innate responses by enhancing the adaptive immune response [25]. However, insufficient adaptive immune reaction, waning immunity, and immune escape can undermine or circumvent the sterilizing characteristics of immunity and allow for subsequent reinfection [26]. In this study, the relative cytokine secretion of IL-4 (60-fold change in the *p*E2-fe/Gel02 cohort vs. 100-fold change in the strain C cohort at 31 dpi, *p* < 0.001), IFN-γ (58-fold increase in the *p*E2-fe/Gel02 cohort vs. 82-fold increase in the strain C cohort at 31 dpi, *p* < 0.001), and PBMC proliferation index (SI of 0.4 in the *p*E2-fe/Gel02 cohort vs. 0.6 in the strain C cohort at 41 dpi, *p* < 0.001) illustrated that the E2-ferritin nanoplatform may simultaneously elicit both humoral and cellular immunity. Therefore, structure- and function-based pathogen design should focus on the activation of adaptive immunity.

At 14 dpc, all rabbits were euthanized and subjected to CSFV-related pathological detection. None of the rabbits in any of the cohorts showed gross pathological changes. However, rabbits immunized with PBS, pfe, and live-attenuated C-strain showed slightly enlarged spleens and lymph nodes, whereas the other rabbits in corresponding cohorts were lesion-free. From this perspective, E2-ferritin nanocage vaccines are proven to be safe and are worth promoting. Infectious viral pathogen-interrelated nanovaccines and self-assembling ferritin nanocages are also being developed in therapeutic anticancer nanomedicines, such as a targeted ferritin nanoparticle that inhibits tumor growth via a novel cancer immunotherapy strategy of encapsulating CpG oligodeoxynucleotides [33].

Though the viremia in ATP was considered as an optional evaluation index, the results showed that the E2-ferritin nanocage cohorts could effectively eliminate the viremia symptoms (less than 331.7 copies/μg total RNA) that were caused by live CSFV or live-attenuated vaccines. Analogously, although there was no statistical difference among the E2-interrelated vaccine cohorts, the full-dose (40 μg) cohort of *p*E2-fe/Gel02 has the lowest virus copy numbers (148 lgCopies/μg total RNA in spleen and 165 lgCopies/μg total RNA in blood), which indicated that there was maximum virus clearance occurring in the *p*E2-fe/Gel02 cohort. In addition, the viral copy numbers in the half-dose (20 μg) cohort of E2-fe/Gel02 (221 lgCopies/μg total RNA in spleen and 177 lgCopies/μg total RNA in blood) were equivalent to that of the full-dose (40 μg) ferritin-free cohort of *p*E2/Gel02 (211 lgCopies/μg total RNA in spleen and 182 lgCopies/μg total RNA in blood), which uncovered a cost-efficient vaccine development strategy (Table S1).

## 5. Conclusions

In this study, we successfully applied the protein-based self-assembling ferritin nanoplatform into the design of CSFV nanovaccines using BEVS.

Specially, the in vivo results demonstrated that rabbits immunized with ferritin nanoparticle vaccines of *p*E2-fe (2/4), *p*E2-fe/Gel02 (3/4), and E2-fe/Gel02 (2/4) were earlier than the subunit vaccine *p*E2/Gel02 (1/4) in developing E2-blocking antibodies at 11 dpi. Importantly, the results from the neutralizing antibody titration strengthened the effectiveness of this nanoplatform vaccine in neutralizing antibody induction. Thus, this novel nanovaccine could elicit a robust humoral immune response compared to the traditional subunit vaccines in the ATR model.

Evidently, the viral clearance in the full-dose (40 μg) cohort *p*E2-fe/Gel02 and the half-dose (20 μg) cohort of E2-fe/Gel02 post viral challenge uncovered a novel and cost-efficient vaccine development strategy. The relative RT-qPCR analysis of IL-4 and IFN-gamma illustrated that the E2-ferritin nanoplatform may simultaneously activate both humoral and cellular immunities.

In conclusion, the approach and systems depicted here could be expanded to other nanovectors, such as polymer and inorganic nanoparticles, as well as a variety of protein-based nanocages carrying various immunogens, offering a novel paradigm for potent neutralizing antibody-based vaccine development and efficient T cell immunity induction against some infectious viral diseases.

## Figures and Tables

**Table 1:** Primers for ferritin nanoplatform construction and RT-qPCR.

Primers	Sequences (5′–3′)	Products
CSFV-E2	Forward: GGTACCCGGCTAGCCTGCAAGGAAG	1159-bp
	Reverse: ACGCGTTTAATGATGATGATGATGATGTTCTGCGAAGTAATC	
RT-qPCR (5′UTR/strain C)	Forward: GAACTGGGCTAGCCATG	98-bp
	Reverse: ACTGTCCTGTACTCAGGAC	
	Probe: FAM-TAGGACTAGCAAAACGGAGGGACTAGCCA-TAMARA	
GAPDH (rabbit)	Forward: AGAGCACCAGAGGAGGACG	108-bp
	Reverse: TGGGATGGAAACTGTGAAGAG	
IFN-gamma (rabbit)	Forward: CTGGTCCAGCGTAAAGCAGT	116-bp
	Reverse: TCAGTACTTGGATGCTCGCC	
IL-4 (rabbit)	Forward: CAGGGGCGACATCATCCTAC	102-bp
	Reverse: CTCGGTTGTGTTCTTGGGG	

**Table 2:** Immunization procedure and dosages in the ATR evaluation model.

Cohort (*n* = 4)	Dosage	Antigens	Immunization	Challenge	Sacrifice (56 dpi)
PBS	1 mL (IM)		Prime vaccination: Day 1; Booster vaccination: Day 21; Intramuscular injection (IM)	Viral challenge: C-strain (1 dose) Intravenous injection (IV)	Spleen collection for viral tissue load assay
*p*fe	40 μg (IM)	40 μg purified			
*p*E2-fe	40 μg (IM)	40 μg purified			
*p*E2-fe/Gel02	40 μg (IM)	40 μg purified			
E2-fe/Gel02	40 μg (IM)	20 μg (unpurified)			
*p*E2/Gel02	40 μg (IM)	40 μg purified			
C-strain	1-dose (IV)	1-dose			

## Data Availability

The data presented in this study are available on request from the corresponding author.

## Supplementary Materials

The following are available online at https://www.mdpi.com/2076-393X/9/1/45/s1, Figure S1: The construction of different forms of vaccines. The truncated CSFV C-strain (GenBank accession no. AY805221.1) glycoprotein E2 without signal peptide sequence and transmembrane region (residues 21-341), and H. Pylori ferritin (GenBank accession no. NP_223316, residues 5-167) with a point mutation (N19Q) to abolish a potential N-linked glycosylation site were synthesized by Sangon Bio (Shanghai, China) (up and middle) [34,35]. Considering that the formation of ferritin nanocage, the flexible G-S-G linker was fused to eliminate the interference of glycoprotein E2 on the formation of ferritin nanoplatform (down). The Sp (signal peptide, human CD5 leader sequence) was fused to the N-terminal to generate a secreted protein. All the codon sequence was modified by insect cell expression vector system. Figures S2–S5: The whole blot (uncropped blots) showing all the bands with all molecular weight markers of Figure 2a,b,e,f. Table S1: LgCopies of CSFV/μg total RNA read in Figure 5b,c.
