# Supplementary material for: A Self-Assembling Ferritin Nanoplatform for Designing Classical Swine Fever Vaccine: Elicitation of Potent Neutralizing Antibody
Source: Vaccines (Basel). 2021 Jan 13;9(1):45. doi: 10.3390/vaccines9010045 (PMC7828615; doi:10.3390/vaccines9010045)
Supplement: Supplementary file 1 [file vaccines-09-00045-s001.pdf]

Supplementary materials

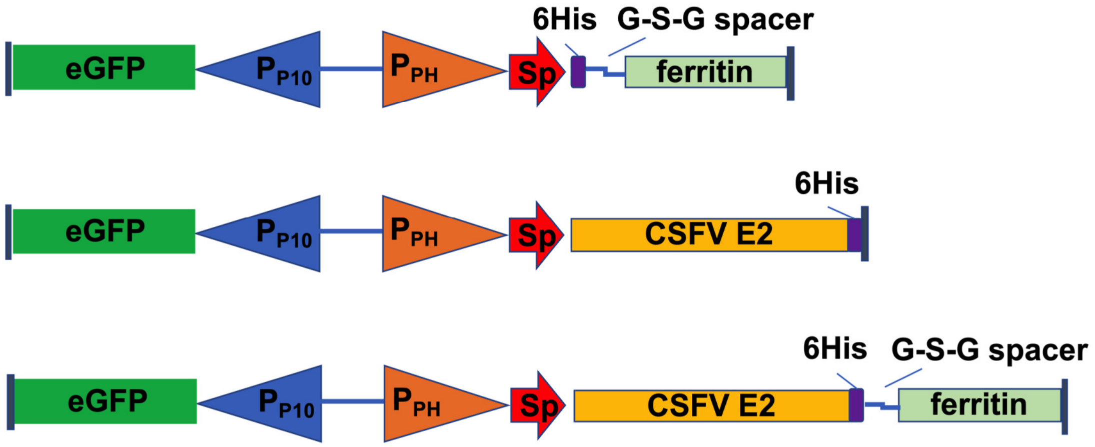

Figure 1. The construction of different forms of vaccines of figure 1a is.

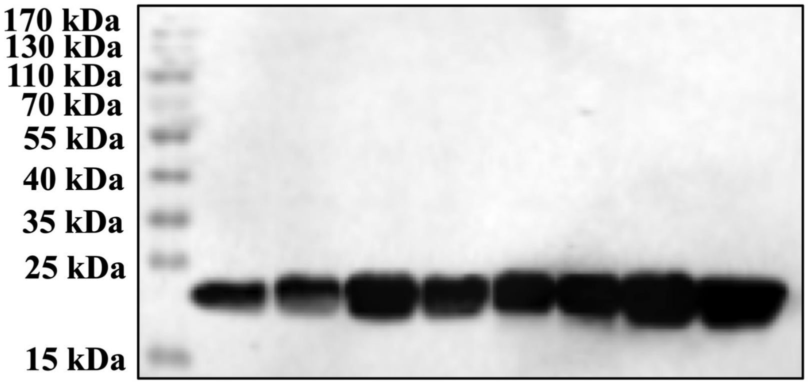

Figure 2. The whole blot (uncropped blots) showing all the bands with all molecular weight markers of figure 2a is.

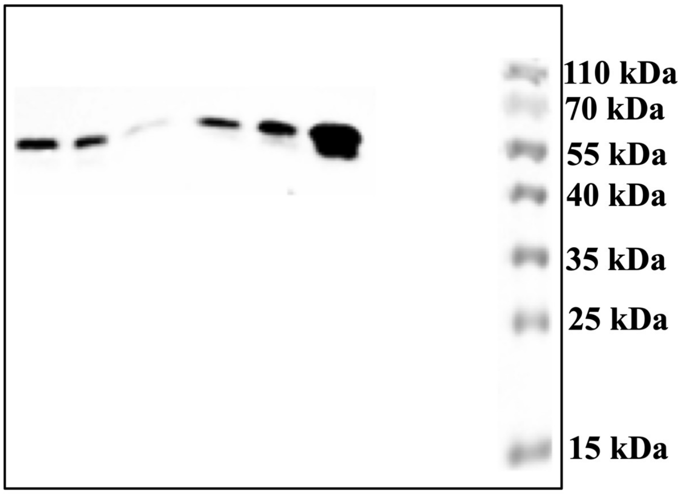

Figure 3. The whole blot (uncropped blots) showing all the bands with all molecular weight markers of figure 2b is.

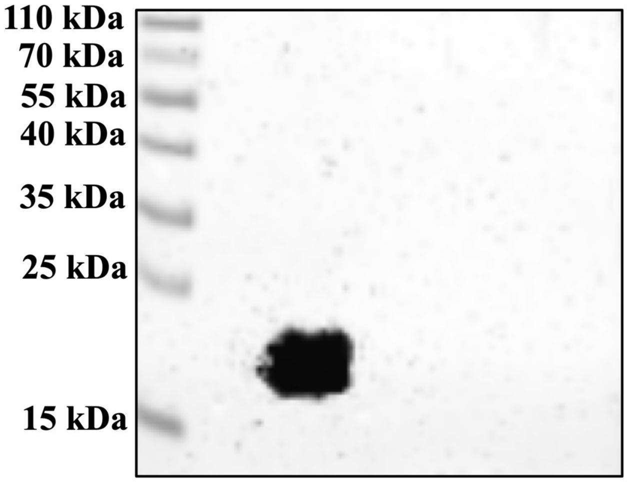

Figure 4. The whole blot (uncropped blots) showing all the bands with all molecular weight markers of figure 2e is.

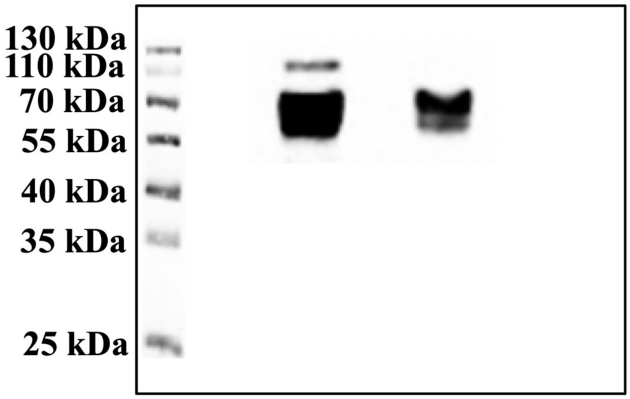

Figure 5. The whole blot (uncropped blots) showing all the bands with all molecular weight markers of figure 2f is.

Table S1. LgCopies of CSFV/μg total RNA read in Figure 5b and 5c is.

Cohort	Viral spleen load	Mean 1 (spleen)	Viremia	Mean 2 (viremia)
PBS	689.9; 512.2; 1294.9; 550.4;	761.85	542.9; 592.1; 396.8; 457.9;	497.425
*p*fe	560.5; 542.6; 1178.9; 391.6;	668.4	465.5; 344.6; 368.3; 493.6;	418
*p*E2-fe	280.2; 199.9; 241.2; 228.1;	237.35	146.3; 136.4; 225.6; 329.5;	209.45
*p*E2-fe/Gel02	145.2; 183.6; 131.9; 132.4;	148.275	117.4; 189.3; 139.1; 213.9;	164.925
E2-fe/Gel02	190.4; 231.5; 178.1; 284.2;	221.05	183.2; 131.6; 213.1; 178.2;	176.525

*p*E2	228.1; 310.6; 148.9; 156.3;	210.975	239.1; 221.4; 118.3; 149.3;	182.025
C-strain	210.7; 179.4; 148.3; 230.7;	192.275	129.7; 219.3; 308.1; 170.3;	206.85
